# Molecular epidemiology, genetic variability and evolution of HTLV-1 with special emphasis on African genotypes

**DOI:** 10.1186/s12977-019-0504-z

**Published:** 2019-12-16

**Authors:** Philippe V. Afonso, Olivier Cassar, Antoine Gessain

**Affiliations:** Unité d’Epidémiologie et Physiopathologie des Virus Oncogènes, CRNS-UMR 3569, Département de Virologie, Institut Pasteur, Bâtiment Lwoff, 28 rue du Dr. Roux, 75724 Paris cedex 15, France

**Keywords:** HTLV-1, Molecular epidemiology, Evolution, Africa, Genotypes, Mutation rate

## Abstract

Human T cell leukemia virus (HTLV-1) is an oncoretrovirus that infects at least 10 million people worldwide. HTLV-1 exhibits a remarkable genetic stability, however, viral strains have been classified in several genotypes and subgroups, which often mirror the geographic origin of the viral strain. The Cosmopolitan genotype HTLV-1a, can be subdivided into geographically related subgroups, e.g. Transcontinental (a-TC), Japanese (a-Jpn), West-African (a-WA), North-African (a-NA), and Senegalese (a-Sen). Within each subgroup, the genetic diversity is low. Genotype HTLV-1b is found in Central Africa; it is the major genotype in Gabon, Cameroon and Democratic Republic of Congo. While strains from the HTLV-1d genotype represent only a few percent of the strains present in Central African countries, genotypes -e, -f, and -g have been only reported sporadically in particular in Cameroon Gabon, and Central African Republic. HTLV-1c genotype, which is found exclusively in Australo-Melanesia, is the most divergent genotype. This reflects an ancient speciation, with a long period of isolation of the infected populations in the different islands of this region (Australia, Papua New Guinea, Solomon Islands and Vanuatu archipelago). Until now, no viral genotype or subgroup is associated with a specific HTLV-1-associated disease. HTLV-1 originates from a simian reservoir (STLV-1); it derives from interspecies zoonotic transmission from non-human primates to humans (ancient or recent). In this review, we describe the genetic diversity of HTLV-1, and analyze the molecular mechanisms that are at play in HTLV-1 evolution. Similar to other retroviruses, HTLV-1 evolves either through accumulation of point mutations or recombination. Molecular studies point to a fairly low evolution rate of HTLV-1 (between 5.6E−7 and 1.5E−6 substitutions/site/year), supposedly because the virus persists within the host via clonal expansion (instead of new infectious cycles that use reverse transcriptase).

## Background

The human T-cell lymphotropic virus (or T-cell leukemia virus) type 1 (HTLV-1), discovered in 1980, has been identified as the first human oncoretrovirus [1]. HTLV-1 is a member of the *Retroviridae* family, the *Orthoretrovirinae* subfamily and the Deltaretrovirus genus, which includes bovine leukemia virus (BLV) and T-lymphotropic viruses infecting primates (PTLV). PTLVs consist of simian T-lymphotropic viruses (STLVs) type 1 to 4, which infect non-human primates and human T-lymphotropic viruses type 1–4.

HTLV-1 is the etiological agent of two main very severe diseases: a lympho-proliferative disorder, of mainly CD4 T-cells, named adult T-cell leukemia/lymphoma (ATL) [2], and a chronic neuromyelopathy named tropical spastic paraparesis/HTLV-1 associated myelopathy (TSP/HAM) [3, 4]. HTLV-1 is also associated with other inflammatory diseases including infective dermatitis, some forms of uveitis, myopathies, and bronchiectasis [5].

At least 5 to 10 million people are infected with HTLV-1 worldwide. The known high endemic areas for HTLV-1 are Southwestern Japan, the Caribbean region, parts of South America, sub-Saharan Africa, some foci in the Middle East, and Australo-Melanesia [6–8]. The origin of this puzzling geographical (and often ethnic) repartition is likely related to a founder effect in isolated groups where elevated viral transmission rate have persisted. HTLV-1 transmission occurs through sexual intercourse, prolonged breast-feeding, or blood transfusion. Upon leukoreduction, HTLV-1 transmission during transfusion is reduced, evidencing the importance of cell-associated virus in this case [9, 10]. HTLV-1 seroprevalence increases with age, is usually higher in women, and reaches 40% in some highly endemic areas [6–8, 11].

## HTLV-1 genotypes: classification and geographical distribution

The first HTLV-1 complete sequence (ATK prototype) was obtained in 1983 [12]. It originated from a Japanese patient with ATL. In the following years, many sequences were generated and revealed low genetic variability [13–16]—when compared to HIV-1 for instance [17]. Interestingly, no evidence for a specific mutation associated with TSP/HAM or ATL was found. In contrast, some nucleotide substitutions observed among HTLV-1 strains were specific to the geographic origin of the patients [18].

**Fig. 1:**
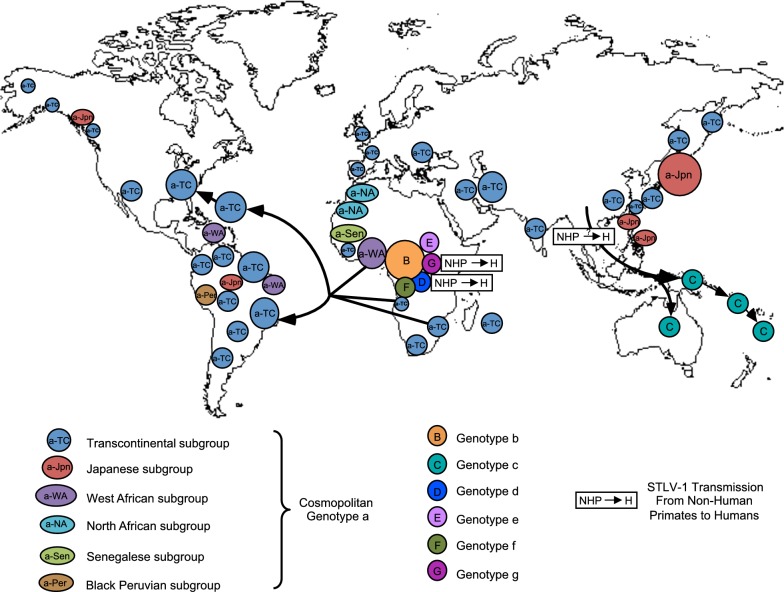
Geographical distribution of the seven main molecular genotypes of HTLV-1 (a–g) and major pathways for the spread of the virus through the movements of infected populations

**Fig. 2:**
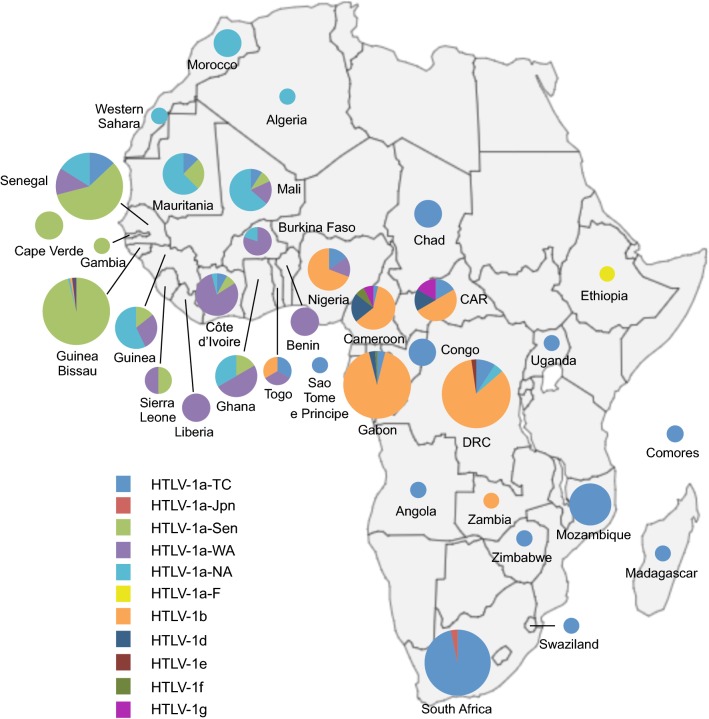
Map of Africa showing the general distribution of HTLV-1 genotypes across the continent. The proportion of the different HTLV-1 genotypes and subgroups is presented for each African country. This figure incorporates the information from papers of molecular epidemiology available on PubMed [20, 21, 23–27, 30, 41, 44–46, 55, 68, 135–144]. It also incorporates results from two manuscripts in preparation (Cassar et al. and Filippone et al.), notably concerning the situation in Benin, Sierra Leone, Western Sahara, and Madagascar, where no data were available to our knowledge. Countries without indications have no informative published data on HTLV-1 genotypes between 1994 and 2019. The size of the circles is proportional to the number of strains identified. The smallest size corresponds to 1 characterized strain, the intermediate sizes to a maximum of 5 or 29 strains and the largest to a minimum of 30 strains. HTLV-1a-North African (HTLV-1 a-NA), HTLV-1a-Senegalese (HTLV-1 a-Sen), HTLV-1a-West African (HTLV-1 a-WA), HTLV-1b and HTLV-1a-Transcontinental (HTLV-1 a-TC) are the most common throughout the continent in North, West, Central and the Austral parts respectively. HTLV-1 d, -e, -f and-g have been identified in Central Africa (Cameroon, Central African Republic, and Gabon)

**Fig. 3:**
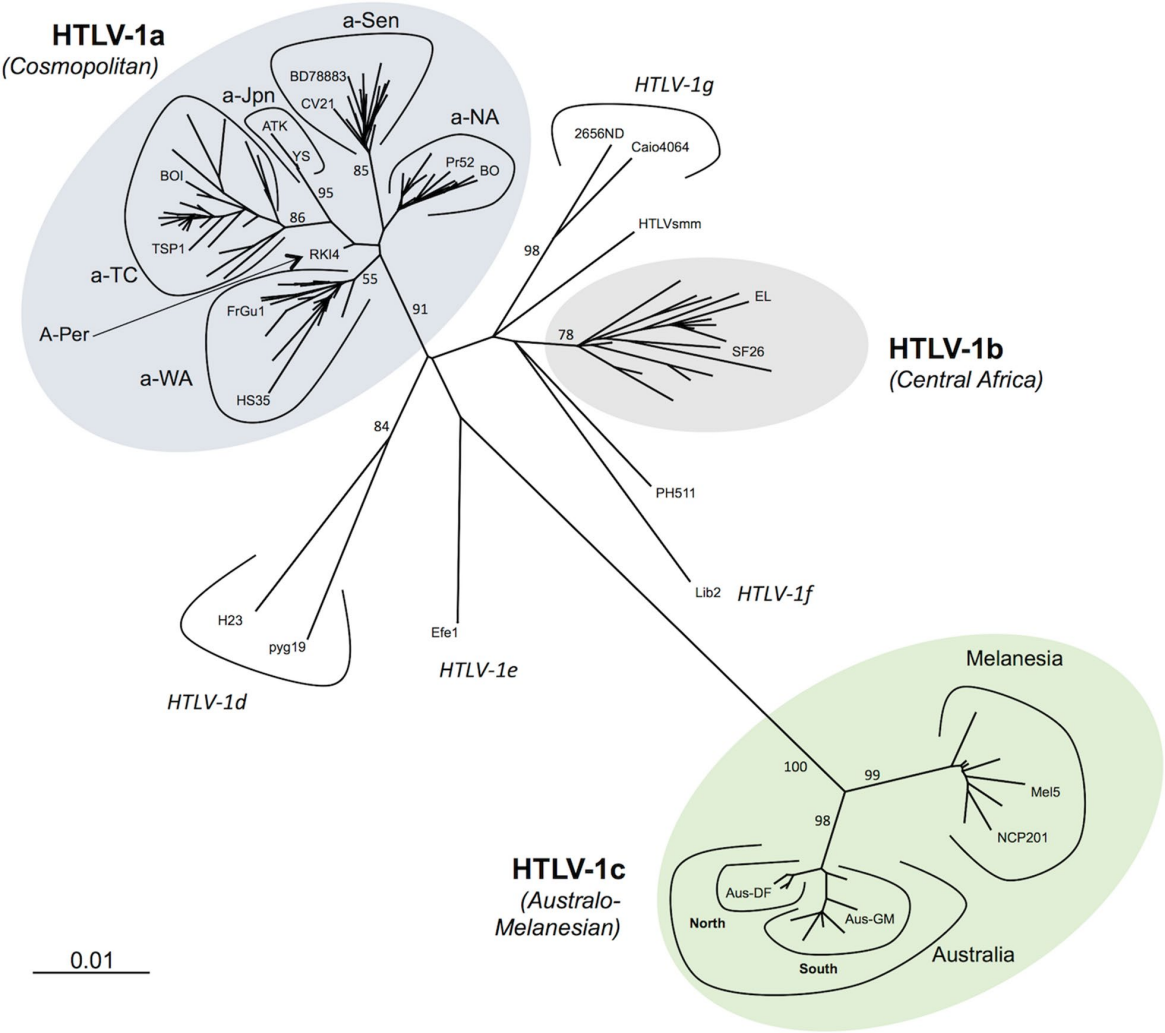
Phylogenetic representation of the HTLV-1 genotypes and subgroups. An alignment of complete LTR sequences (774-nt long) from 178 HTLV-1 strains was obtained. The unrooted phylogenetic tree was generated with the neighbor-joining method using the GTR model (gamma = 0.4953). Branch lengths are drawn to scale, with the bar indicating 0.01 nucleotide replacement per site. Numbers on each node indicate the percentage of bootstrap samples (of 1000 replicates). HTLV-1 genotypes (a–g) and subgroups (within HTLV-1a and HTLV-1c) are presented. References strains (presented in the table) are indicated in the tree, except Mel1 and Ethio10 for which the complete LTR sequence is not available

Three major molecular genotypes (or subtypes) have been successively identified: the Cosmopolitan a-genotype, the Central African b-genotype, and the Australo-Melanesian c-genotype (Table 1, and Figs. 1 and 2). Other minor genotypes have also been characterized in Central Africa: genotypes -d, -e, -f and -g (Table 1, and Figs. 1, 2, 3) [6, 8]. There is no definite rule for the definition of each genotype, but each genotype is supported by phylogenetic studies (Fig. 3), and intragenotypic variability is lower than intergenotype variability.

**Table 1:** Reference sequences for the different HTLV-1 genotypes and subgroups

Genotype	Subgroup	Reference strains	Country of origin	Genbank number	Refs.
a	A/Transcontinental (a-TC)	BOI	France	L36905	[13]
		TSP1	Japan	M86840	[16]
	B/Japanese (a-Jpn)	ATK	Japan	J02029	[12]
		YS	Japan	U19949	[145]
	C/West African (a-WA)	HS-35	Caribbean	NC001436	[14]
		FrGu1^a^	French Guyana	AY324785	[98]
	D/North African (a-NA)	BO^a^	Algeria	U12804	[21]
		Pr52^a^	Morocco	U12806	[21]
	E/Black Peruvian (a-Per)	Bl1^a^	Peru	Y16481	[22]
		RKI4^a^	Peru	AF054627	[22]
	F	Ethio10^a^	Ethiopia	KC493410	[23]
	Senegalese (a-Sen)	BD78883^a^	Senegal	DQ235700	[26]
		CV21	Cabo Verde	KX430030	[136]
b		EL^a^	Africa	M67514	[146]
		SF26	Brazil	JX507077	[141]
c	Melanesia	Mel5	Solomon Islands	L02534	[50]
		NCP201	New Caledonia	KX905203	[52]
	Australia	Aus-DF	Australia	KF242505	[53]
		Aus-GM	Australia	JX891478	[53]
d		Pyg19^a^	CAR	L76310	[27]
e		Efe1^a^	DRC	Y17014	[54]
f		Lib2^a^	Gabon	Y17017	[54]
g		2656ND^a^	Cameroon	AY818431	[55]
		HTLVsmm	Liberia	KU214243	[147]

Two strains have been proposed as reference strains for each subgroup within genotypes a and c, and for genotype b. When available, complete sequences are presented. Otherwise, historic strains are presented. Due to the limited number of available strains for genotypes d–g, a single strain is presented. Of note, the complete sequence HTLVsmm does not belong to a characterized human genotype

GenBank number corresponds to either the complete sequence, or alternatively the LTR sequence. For each strain, the genotype (and subgroup) is presented, together with the country of origin. The letter in the subgroup section corresponds to the historic name of the group; the second element corresponds to the current denomination of the subgroup

^a^Partial sequences (The GenBank number corresponds to the complete or partial LTR sequence)

The Cosmopolitan a-genotype is the most frequently reported clade and is distributed worldwide. Indeed, it is present in various areas such as Japan, the Caribbean region, Central and South America, West and South Africa, the Middle East, and Europe. This genotype can be further divided into geographically related subgroups. Subgroups are monophyletic clades that can emerge within a genotype, but inter-subgroup genetic diversity is low thus it cannot be considered as a genotype per se. The existence of subgroups suggests that viruses have spread with the migration of ancient infected populations, and have been genetically isolated for centuries or thousands of years.

The initial classification comprised the Transcontinental A subgroup, the Japanese B subgroup, the West-African C subgroup, and the North African D subgroup; they are now referred as a-TC, a-Jpn, a-WA, and a-NA, respectively (Table 1, and Fig. 3) [19–21]. More recently, the E/a-Per subgroup, comprising 2 strains from Black Peruvian, was defined [22]; based on partial segment of LTR, a F subgroup has also been identified, especially in an Ethiopian patient [23]. Lastly, we have added in 2006, a Senegalese subgroup (a-Sen), which has also been named “Trans-Saharan” or clade W within the HTLV-1aD subgroup [24–26].

**Fig. 4:**
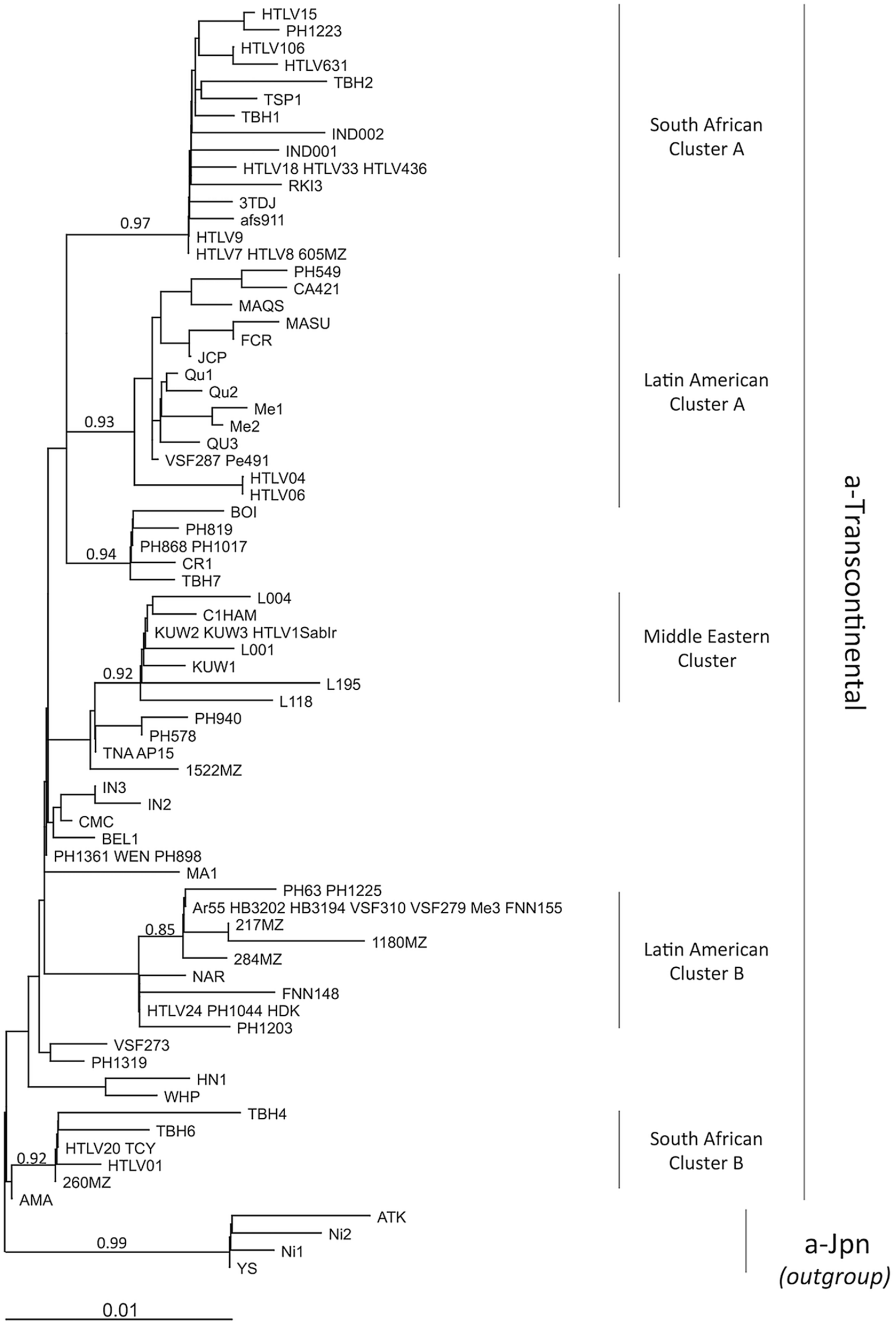
Diverse clusters can be identified within the HTLV-1a-TC subgroup. An alignment of LTR sequences (519-nt long) from 91 HTLV-1a-TC strains was obtained. Sequences from HTLV-1a-Jpn were used as outgroup. The phylogenetic tree was generated with the neighbor-joining method using the GTR model (gamma = 0.4953). Horizontal branch lengths are drawn to scale, with the bar indicating 0.01 nucleotide replacement per site. Values correspond to the approximate likelihood-ratio test for each clade
- In Japan, strains from the a-TC subgroup coexist with Japanese specific strains [35–39]. The ratio between these two subgroups differs depending on geographical areas and local populations. While the a-TC subgroup is highly predominant among the Ainu in the North and residents of Okinawa (Southwest Japan), the a-Jpn subgroup is predominant among the Wajin population in mainland Japan. Ryukyuans are infected with strains of both subtypes. The reasons for such ethnic and geographical distribution are still under debate. It is believed that the ancestors of the Wajin population were infected when arriving in Japan, and that this virus then evolved into the HTLV-1 a-Jpn. HTLV-1 a-TC may have been introduced more recently in Japan.
- In Côte d’Ivoire and Ghana, the majority of HTLV-1 strains belong to the West African subgroup (Fig. 2) [40, 41]. a-WA strains were also introduced in South America via the Slave Trade: a-WA strains are found among the Noir-Marron populations living in French Guiana and Surinam [42]. Noir-Marrons are descendants of the slaves who escaped from plantations in the Dutch colony of Surinam during the sixteenth and early seventeenth century. The Noir-Marron have strong genetic affinities close to African populations from the bight of Benin, which is consistent with their predominant HTLV-1 genetic subtype [29, 43].
- The Senegalese subgroup represents, by definition, the major subgroup present in Senegal (Fig. 2) [25, 26]. It is also present in neighboring countries such as Gambia, Guinea-Bissau, and Mali [24, 44]. In addition, the a-Sen strains are found, but more rarely, in Côte d’Ivoire and Ghana. This is probably a testimony of frequent migrations, some still ongoing, of people from Senegal and neighboring countries to other parts of West Africa.
- The North African subgroup is mainly present in Algeria, Morocco, Mauritania, Western Sahara, and Mali (Fig. 2) [21, 41]. It can also be found sporadically in other West African countries such as Senegal, Guinea, Côte d’Ivoire, and Ghana.

- The transcontinental (TC) subgroup is present on all continents. The overall nucleotide variability within subgroup a-TC is low: it can reach 0–2.5% in the gp21-*env* gene and 0–2% in the LTR region [27]. It is believed that this low genetic variability reflects the recent dissemination of these strains. In particular, the slave trade from Africa to America, which peaked in the eighteenth century, may represent one of the major paths of recent dissemination [22, 28, 29]. Indeed, HTLV-1 strains found in South Africa, Mozambique, Zimbabwe, Swaziland, and Angola cannot be distinguished from strains found in Brazil [6, 7, 30–32]. Additionally, in some studies, clades within the a-TC subgroup have been identified such as South African clusters, Latin-American clusters, and a Middle Eastern cluster [22, 33, 34] (Fig. 4).

The Central African b-genotype is most frequently found in Central Africa, i.e. Cameroon, Gabon, CAR, DRC, and Nigeria (Fig. 2). It represents more than 90% of the strains found in Gabon and DRC [27, 45, 46]. HTLV-1b strains differ from HTLV-1a by 2–3% at the nucleotide level (compared to the ATK reference strain) [27]. As for HTLV-1a, strains can cluster according to the geographical origin: HTLV-1 strains from DRC are closer to each other than to strains found in South Cameroon and Gabon, for example [46].

The Australo-Melanesian c-genotype is the most divergent: the genetic nucleotide variability can reach 6–9% when compared to the reference ATK prototype. This reflects an ancient speciation, with a long period of isolation of infected populations living in the different islands of this Pacific region. HTLV-1c was first described in a small group of hunter-horticulturalists living in the fringe highlands of Papua New Guinea (PNG) [47–49] and among people of Melanesian origin living in the Solomon Islands [49, 50]. Since, HTLV-1c strains have also been found among residents from Central Australia, the Vanuatu Islands, and New Caledonia [51–53]. As with other genotypes, genetic clades that mirror geography can be identified within the HTLV-1c genotype. Phylogenetic analyses indicate the existence of a subgroup composed of strains from the Solomon Islands, the Vanuatu archipelago and New Caledonia (Melanesia subgroup), on the one hand, and an Australian subgroup, on the other (Fig. 3). The Australian subgroup can be further subdivided into two clades (North and South) [53] (Fig. 3).

Other genotypes d, e, f and g have been reported in Central Africa, mainly in Cameroon, Gabon, DRC and CAR [27, 54, 55] (Table 1, and Fig. 2). HTLV-1d can represent up to 3% of the HTLV-1 strains in this region [45]; HTLV-1 e–g strains have been reported sporadically.

## The genetic organization differs according to molecular genotypes

HTLV-1 is a complex retrovirus: in addition to structural and regulatory proteins, it encodes several accessory proteins (also called auxiliary proteins). In the HTLV-1a genome, two open reading frames (ORFs) encode four accessory proteins: p12 is encoded by ORFI and can be cleaved into p8; p13 and p30 are encoded by ORFII and are obtained by alternative splicing [56]. These proteins display functions essential for viral persistence in vivo: p12 facilitates immune escape, p8 enables viral propagation, p30 is a negative regulator of viral replication and favors viral persistence, and p13 modulates the cellular response to oxidative stress and allows infected cells to survive [57, 58]. It was early found that deletion of accessory ORFs limits the replication capacity of HTLV-1 in animal models [59], although in some cases mutation in the accessory ORF may have led to disruption of the *hbz* ORF. Valeri et al. [60] generated a virus deleted for ORF I, with the conservation of *hbz*. This virus could persist in the rabbit model, but could not persist in Macaques. Thus, the importance of accessory proteins may depend on the host species. Interestingly, some replication may still occur in the latter model as some revertants could appear. Some variability of these accessory proteins has been reported, particularly with regard to p12. Truncated forms of p12 have been described in Japan and South America [61–63]. Moreover, two isoforms at position 88 (R/K), which can be linked to different levels of protein expression and degradation, have also been observed [64]. However, it is unclear whether this diversity has an impact on viral expression and pathogenesis in vivo.

The importance of accessory proteins in vivo has been regularly questioned. A HTLV-1a strain deleted for p12 has been described in 3 siblings, suggesting that this virus was transmitted by their mother and is therefore capable of transmission, replication, and persistence in vivo [61]. Similarly, in closely related BLV, the mutation of accessory proteins (R3 and G4) attenuates the virus; the attenuated virus can still replicate and, in the long term, cancers can still appear (although rarely) [65]. Finally, a recent in silico study comparing the complete PTLV-1 genomes available on GenBank confirmed, as expected, that each complete HTLV-1a strain has accessory ORFs and encodes for the 4 proteins. In contrast, strains of the HTLV-1c and -1b subtypes lack some accessory genes [66]. The start codon of ORF I is missing from the complete HTLV-1c and HTLV-1b sequences. Moreover, the splicing acceptor required to generate the mRNA encoding p30 is mutated and may not be functional.

The absence of accessory ORFs, as suggested in the in silico analysis, may indicate that: (1) the encoded proteins are not essential for viral replication in vivo, (2) there are compensatory mutations in the HTLV-1b and HTLV-1c genomes that turn accessory proteins optional, or (3) there are alternative accessory proteins for these viral subtypes. The latter hypothesis is the most likely. Indeed, although the start codon is absent from the ORFII, the ORF contains no additional stop codon. This may suggest a selective pressure to keep the ORF open. The Franchini’s laboratory recently suggested that alternative splicing could lead to the synthesis of p16, an alternative protein to p12 (personal communication). In conclusion, the genetic organization and accessory genes may be different between viral genotypes.

## HTLV-1 originates from its simian counterpart through interspecies transmission

Many non-human primates (NHPs) are endemic for STLV-1, the simian counterparts of HTLV-1: STLV-1 can be found in chimpanzees, gorillas, mandrills, baboons, several species of African monkey, a wide range of macaques, and orangutans [67–74]. Clonal proliferation of STLV-1 infected CD4 T-cells has been reported in many NHP species [75]. ATLs have also been reported in a series of STLV-1 infected NHPs [76–78].

Interspecies transmission can occur, and is currently ongoing in Central Africa. STLV-1 can be transmitted to humans through infected body fluids, such as saliva and blood. Epidemiological studies have recently found that a severe bite by an ape or a monkey is a major risk factor for HTLV-1 infection in NHP hunters (especially Pygmies) in West Central Africa [79, 80].

It is thus believed that the different HTLV-1 genotypes have originated from ancient interspecies transmission of STLV-1. It is supported by the fact that STLV-1 infecting chimpanzees and gorillas in South Cameroon cannot be distinguished from HTLV-1b strains [80–82]. Similarly, STLV-1d is endemic in Mandrills and *C. nictitans* in Central Africa [67, 70, 73], and STLV-1e and -f are detected in monkeys in Cameroon [67, 83].

However, the case is different for HTLV-1a and -1c. There is no known STLV-1 closely related to these two human genotypes. For HTLV-1a, it can be assumed that either the simian reservoir has not been described yet, or the simian ancestors may have disappeared since the virus was transmitted to humans. For HTLV-1c, the case is even more complex. Indeed, monkeys have never been present in the Australo-Melanesian region. As a result, interspecies transmission of STLV-1 to humans could not occur on these islands. Therefore, it is proposed that HTLV-1c was acquired by proto-Australo-Melanesians during their migration through Southeast Asia, and that populations that reached the highlands of Papua New Guinea were already HTLV-1 infected. The infected populations would then have disseminated, together with their virus, throughout the Australo-Melanesian region [50, 53, 84–87].

In Asia, STLV-1 is found in many species of macaques [69, 74]. Macaque STLV-1 forms a paraphyletic clade composed of genetically very distant strains [66]. These strains are so distinct that some authors have considered that STLV-1 found in *macaca artoides* could constitute a novel genotype, called STLV-5 [88]. Intriguingly, zoonotic transmission of STLV-1 has never been reported in Asia, despite a high endemicity of STLV-1 among macaques, and frequent contacts between monkeys and humans in Asia (as evidenced by the transmission of other retroviruses, such as Foamy virus [89, 90]. The reasons for such an apparent restriction of Asian STLV-1 in humans remain unknown. We have recently speculated that STLV-1 from macaques do not express any accessory proteins necessary for viral persistence in the human host [66].

## Mechanisms of evolution of HTLV-1

Both recombination and point mutations contribute to the genetic variation of retroviruses. However, until recently, recombination was disregarded when considering HTLV-1 evolution. Indeed, no recombination event had been identified for HTLV-1. The absence of recombination was supported by the fact that no superinfection at the cellular level had been described [91]. Recently, we have identified the first recombinant HTLV-1 strains [41]. We have found that some strains collected from individuals in North Africa (a-NA) are the result of a recombination between HTLV-1 strains related to strains currently present in Senegal (a-Sen) and West Africa (a-WA) (Fig. 3). The recombination site was located at the U3-R junction, suggesting that the recombination event may have occurred during reverse transcription (RT). Ongoing studies have confirmed such findings and identified other recombinants among HTLV-1 strains from West and North Africa. (Cassar et al. in preparation). However, we assume that recombination may be a rare event for HTLV-1, and that the main evolution mechanism for HTLV-1 would be the accumulation of point mutations.

Some intra-individual viral genetic diversity has been reported. Ehrlich et al. [92] found, when studying a 173 bp-long fragment of *env*, that 16 of the 19 samples displayed genetic variants. Many mutations could be linked to cytidine deaminase activity. Apart from the G>A transition, 7 samples (out of 19) were composed of multiple strains, suggesting the presence of HTLV-1 quasi-species (or multiple infection).

The origin of such diversity is often attributed to the RT. Indeed, the mutation rate of HTLV-1 RT is estimated at 7E−6 mutation/site/replication cycle [93], which is quite comparable to HIV-1 RT. The magnitude of the mutation spectrum in HTLV-1 patients is much lower than what is reported for HIV-1 [94], which is often related to the fact that the virus propagates in vivo mainly by clonal expansion. Indeed, RT is mainly limited to primo-infection in HTLV-1 [95]. Consistently, mutations introduced by cellular polymerase are limited, at least in asymptomatic carriers. Gessain et al. [28] followed infected individuals overtime and found no change in the viral sequences (i.e. 522 nt-long *env* segment). Of note, the authors had followed only 3 individuals for 6 to 20 months, which explain why no mutation emerged. However, by studying the viral genetic diversity within (and between) infected cellular clones, Mortreux et al. [96] suggested that actually most of the mutations found in the samples were still accumulated during clonal expansion, instead of RT.

In a nutshell, the origin of intra-individual genetic diversity is mostly related to genetic instability and mutations that occur during proliferation of infected cells.

## HTLV-1 evolution rate and molecular clock

There are two different methods for estimating the evolution rate of HTLV-1. Such an estimate only takes into account single point mutations, and recombinant strains should be excluded.

On the one hand, the mutation rate can be estimated by studying vertical/intrafamilial transmission chains of the virus. In this context, remarkable genetic stability was observed: first, a study in the DRC (ex-Zaïre) revealed that 10 related individuals carried the same virus, without mutation (in a 755-nt segment of the LTR), although one member was also co-infected with a second strain that differed in one nucleotide [97]. This latter was either the result of a secondary infection, or a mutation that had occurred in that particular individual. A follow-up study, combining this family together with families from South America, found only two mutations in the LTR (756 bp-long) and three mutations in *env* (522 bp-long) within 16 vertical transmission chains [98]. As a result, mutation rates were estimated at 3.5E−6 and 7.3E−6 substitutions/site/year for LTR and *env*, respectively. In a similar study in Brazil, the estimation was found surprisingly high (2E−5 substitutions/site/year for LTR), supposedly because it was calculated on the basis of 1 mutation on a single mother–child pair [30]. This value may be largely overestimated. Indeed, in Melanesia, the intra-familial genetic heterogeneity is as low as 0–0.2% over 931 nt [99]. This method focuses mainly on vertical transmission of the virus and generates an estimation of the mutation rate in the short time scale.

On the other hand, the mutation rate can be estimated using phylogeny and an anthropological approach, using a dating anchor point for a given clade. Such analyses are based upon several assumptions: (1) the data set is informative, i.e. the genetic variability is not too high and the phylogenetic signal is not saturated. Salemi et al. [100] found that the data set consisting of each codon of the different canonical genes (i.e. *gag*, *pol*, *env*) were informative for studying all PTLVs (PTLV-1–2 and 3). Similarly, when considering PTLV-1 only, the LTR sequences are also informative [101]. (2) The mutation rate is quite comparable between species (HTLV/STLV) and viral types (PTLV-1/2/3). HTLV and STLV are often considered together in the different analyses. Similarly, PTLV-1 and PTLV-2 are often joined in the studies [100–102]. However, it has been shown that HTLV-2 strains isolated from IDUs evolve significantly faster than HTLV-2 strains in an endemic context. Thus HTLV-2 strains from IDUs should be discarded. (3) Either the molecular clock hypothesis is valid or not; in this latter case, a ‘relax clock’ model should be used through Bayesian statistical analysis. The different published papers diverge on this particular point. Salemi et al. [100] found that a data set comprising the 3rd codon of the canonical genes could support the molecular clock hypothesis, when excluding the HTLV-2 IVDU strains. Instead, Lemay et al. [102] preferred studying the 3 codon altogether, and used a Bayesian approach in order to implement a relaxed clock model. When studying HTLV-4, Switzer et al. [88] found saturation on the 3rd codon, and the data set consisting of the 1st and 2nd codon was not suitable with the molecular clock hypothesis. They also had to use a Bayesian approach.

The calibration values for the molecular clock can be major points of debate, and are based on strong assumptions.

The most commonly used date to estimate the time scale for PTLV evolution is the divergence date between HTLV-1c and PTLV1a/b, which is estimated between 40,000 and 60,000 years ago [88, 100–102]. It was at this time that the first populations migrated from Asia to Melanesia. As discussed above, since no simians have ever been detected in Oceania, populations that transmitted HTLV-1 to Australo-Melanesia are considered to have acquired the virus from Indonesian NHPs on their migration route [84]. However, recently, Reid et al. [103] have challenged this dating. They believe HTLV-1 was introduced into Australo-Melanesia much recently, during a more massive wave of migration that originates from India, about 4000 years ago. This change in dating would results in a different and much higher mutation rate.

Another possible date is the divergence between HTLV-2a and -2c (in studies combining the two types of viruses). Indeed, these two clades are composed exclusively of strains present in Amerindian populations. It was therefore proposed that they share a common ancestor who reached the Americas at the time of human migration on the Bering Strait. Thus, the HTLV-2a/c node is dated at 25,000 ± 5,000 years ago [100, 104].

In conclusion, depending on the different models and assumptions, the estimated mutation rates vary from 5.6E−7 [102] to 1.5E−6 [101] and 6.2E−6 [103] subst/site/year, for the LTR. When considering coding regions, the substitution rate is between 2.1E−7 and 8E−7 subst/site/year (assuming a Bayesian relaxed molecular clock) [88, 102].

## Conclusions: major unanswered questions concerning HTLV-1 molecular variety

Despite a good understanding of the genetic diversity and evolution mechanisms of HTLV-1, many questions remain concerning the origins of some groups infected with HTLV-1, and the pathogenicity of each genotype.

1. Several European countries (e.g. France, Great Britain and Spain) regularly report cases of HTLV-1 infection (among blood donors or pregnant women) or HTLV-1 associated diseases [105–107]. In these countries, most of the infected individuals come from regions where HTLV-1 is highly endemic, such as the Caribbean area, sub-Saharan Africa, and South America. In contrast, Romania has a high prevalence of HTLV-1 infection [108, 109], but there is no evidence of significant migrations from HTLV-1 endemicity areas. Thus, Romania seems to be a nucleus of endogenous endemicity in Europe. The origins of HTLV-1 in Romania are unknown. From a molecular point of view, the viral strains present in Romania belong to the TC subgroup of the Cosmopolitan a-genotype [110, 111]. Extensive epidemiological and molecular studies are being conducted in order to get new insights into the origin and dissemination of HTLV-1 infection in Romania.
2. HTLV-1 has been found in many native populations in the Americas, such as the Inuit in Canada and the USA, the Quetchua in Peru, the Mapuche in Chile, and indigenous groups from Argentina [112–115]. Most strains belong to the large a-TC subgroup; in some cases, geographical clusters can be identified (small and large Latin American Clusters, Jujuy specific cluster, etc.) [112–116]. The origin of such infection is still controversial: either the virus has recently been acquired—through contacts with infected individuals from Africa, following the slave trade for example [30–32, 117]—or the virus was introduced during the initial settlement of the American continent, with the migration of infected populations through the Behring Strait [118–120].
3. The modes of dissemination of HTLV-1 in the Middle East and Asia remain to be clarified. Regions of the Middle East (e.g. areas of Iran and Kuwait) have been found endemic for HTLV-1 [121, 122]. A few strains have been characterized, and suggest that there is a Middle Eastern cluster within the HTLV-1a TC subgroup [33, 34, 123]. Interestingly, some strains found in India are closely related to strains from the Middle East [124]. Thus, infected populations have migrated between these regions. Some suggest that the ancient Silk Road, which linked China to Antioch (now in Turkey), could also have been a Road for the dissemination of HTLV-1.
4. The importance of human migrations in the modern area will likely modify the distribution of HTLV-1 and lead to a mixing of genotypes and subtypes. Indeed, the Tokyo metropolitan area may become a hotspot of endemicity for HTLV-1 as individuals migrate from endemic areas such as the Kyushu-Okinawa region [125]. In some cases, long-distance migrations occur and lead to a wider distribution of a previously geographically restricted subtype. Thus, a-Jpn strains have been found in other countries, such as Peru [22], Hawaii USA [126], and South Africa [127] (Fig. 2).
5. There is no clear evidence of specific mutations in the HTLV-1 genome that would render the virus more pathogenic [128, 129]. However, most of the reported cases of ATL and TSP/HAM correspond to individuals infected with HTLV-1 strains from the a-genotype. Does this mean that this genotype is more pathogenic than the others? For instance, it has been suggested that Australian HTLV-1c strains might be less oncogenic, more likely to induce inflammatory diseases (such as bronchiectasis) than tumors [130–132]. Since, ATL cases have been reported in HTLV-1c carriers [133, 134]. One of the reasons why the proportion of ATL appears to be lower among Indigenous Australians may be related to the fact that this population is younger and has a shorter life expectancy; it may also be underreported. In order to clearly answer this particular point, cohort-based prospective studies on HTLV-1b and HTLV-1c populations are needed.

## Data Availability

Not applicable.
